# Aflatoxin, Fumonisin and Shiga Toxin-Producing *Escherichia coli* Infections in Calves and the Effectiveness of Celmanax^®^/Dairyman’s Choice™ Applications to Eliminate Morbidity and Mortality Losses

**DOI:** 10.3390/toxins5101872

**Published:** 2013-10-23

**Authors:** Danica Baines, Mark Sumarah, Gretchen Kuldau, Jean Juba, Alberto Mazza, Luke Masson

**Affiliations:** 1 Lethbridge Research Centre, Agriculture and Agri-Food Canada, 5403 1 Avenue South, Lethbridge, AB T1J 4B1, Canada; 2 Southern Crop Protection and Food Research Centre, Agriculture and Agri-Food Canada, 1391 Sandford Street, London, ON N5V 4T3, Canada; E-Mail: mark.sumarah@agr.gc.ca; 3 PENNSTATE, 321 Buckhout Laboratory, University Park, PA 16802, USA; E-Mail: gak10@psu.edu; 4 PENNSTATE, Fusarium Research Center, 216 Buckhout Laboratory, University Park, PA 16802, USA; E-Mail: jhj2@psu.edu; 5 National Research Council of Canada, Montréal, QC H4P 2R2, Canada; E-Mails: alberto.mazza@cnrc-nrc.gc.ca (A.M.); luke.masson@cnrc-nrc.gc.ca (L.M.)

**Keywords:** Shiga toxin-producing *Escherichia coli*, mycotoxin, prebiotic, probiotic, virulence

## Abstract

Mycotoxin mixtures are associated with Shiga toxin-producing *Escherichia coli* (STEC) infections in mature cattle. STEC are considered commensal bacteria in mature cattle suggesting that mycotoxins provide a mechanism that converts this bacterium to an opportunistic pathogen. In this study, we assessed the mycotoxin content of hemorrhaged mucosa in dairy calves during natural disease outbreaks, compared the virulence genes of the STECs, evaluated the effect of the mucosal mycotoxins on STEC toxin expression and evaluated a Celmanax^®^/Dairyman’s Choice™ application to alleviate disease. As for human infections, the OI-122 encoded *nleB* gene was common to STEC genotypes eliciting serious disease. Low levels of aflatoxin (1–3 ppb) and fumonisin (50–350 ppb) were detected in the hemorrhaged mucosa. Growth of the STECs with the mycotoxins altered the secreted protein concentration with a corresponding increase in cytotoxicity. Changes in intracellular calcium indicated that the mycotoxins increased enterotoxin and pore-forming toxin activity. A prebiotic/probiotic application eliminated the morbidity and mortality losses associated with the STEC infections. Our study demonstrates: the same STEC disease complex exists for immature and mature cattle; the significance of the OI-122 pathogenicity island to virulence; the significance of mycotoxins to STEC toxin activity; and, finally, provides further evidence that prebiotic/probiotic applications alleviate STEC shedding and mycotoxin/STEC interactions that lead to disease.

## 1. Introduction

STEC challenges result in hemorrhagic enteritis (HE) in calves and Jejunal Hemorrhage Syndrome (JHS) in weaned to mature cattle [1,2,3,4]. STEC colonization occurs in the jejunum, ileum, cecum, colon and rectum of immature and mature cattle [3], but attachment and effacement (A/E) lesions have not been found in older calves and mature cattle except in off-trial animals that developed HE during STEC challenge studies [5]. Prebiotic (Celmanax^®^) and probiotic (Dairyman’s Choice™) applications alleviate symptomatic cattle in JHS outbreaks through anti-adhesive behavior that reduces STEC colonization and also by binding mycotoxins [1,2]. The molecular mechanisms underlying differences in STEC pathogenicity for immature calves have been examined using deletion mutants that have deficiencies in the Locus of Enterocyte Effacement (LEE) encoded or non-LEE encoded genes [4]. Intimin and Tir are essential for colonization, A/E formation and development of disease in calves, but are not implicated in eliciting mucosal inflammation [6]. Similarly, Shiga toxins (Stxs) are important for the development of systemic disease in calves [3], but these toxins are not enterotoxic [7,8] and there are no Gb3 receptors present in the vascular system of the colon [9]. There are sub-lethal effects of the Stxs on the mucosa. Stx1 suppresses the activation and proliferation of intraepithelial lymphocytes and macrophages from the mucosa of cattle [6,10], while Stx2 increases STEC colonization of bovine colonic cells *in vitro* [11]. This suggests that there is a role for Stxs in enhancing the expression of HE through an impairment of the intestinal defense system and increasing the ability of STEC to colonize the intestinal tract. Other STEC-secreted toxins have also been implicated in contributing to STEC-associated disease as the amount of cytotoxicity was related to colonization [12]. The composition of STEC infections in natural disease outbreaks support a role for co-infections in the development of serious disease in immature and mature cattle [1,2], but the role of virulence genes in promoting co-infection is unclear. Understanding the composition and genetic nature of STEC infections in cattle is critical to developing a solution for breaking the transmission chain to food products. 

In studies of JHS cases, systemic mycotoxigenic fungal infections were linked to the development of disease [13], but more recent studies support a role for mycotoxins [1,2]. The two dominant mycotoxins associated with JHS cases are fumonisin and gliotoxin, which suppress the immune system *in vitro* [14]. Trichothecenes also cause moderate to severe congestion of the mucosa [15,16]. Cattle exposed to mycotoxin mixtures are colonized by two or more STECs suggesting that mycotoxins are facilitating co-infection [1,2]. If this is true, mycotoxin action may be either direct, such as through greater toxin secretion by the STECs, or indirect, such as altering mucosal integrity or function. There is some evidence that chronic exposure to mycotoxins indirectly affect mucosal integrity where a decrease in the proliferation of undifferentiated epithelial cells alters the integrity of intestinal epithelium thereby facilitating STEC colonization 400 to 700-fold [17]. Since cattle are exposed to various types of mycotoxins via their feed rations, understanding the interactions of mycotoxins with STECs may provide novel insight into how infections are established and maintained.

In this study, we assess the mycotoxigenic fungi and mycotoxins associated with calf starter rations and the transfer of mycotoxins to mucosal tissue for calves that succumb to natural STEC infections. We also characterized the STEC infections to determine if there was a relationship between virulence genes, clinical symptoms and the development of disease. To evaluate the role of mycotoxins in enhancing STEC infection, we assessed the impact of mycotoxins present in the mucosa on the production and activity of STEC-secreted toxins. To our knowledge, this is the first report comparing the detailed virulence gene composition of natural STEC infections in immature calves with experimental infections and the potential role of mycotoxins in mediating infection. It is also further support for the effective use of a combined Celmanax^®^/Dairyman’s Choice™ application to eliminate morbidity and mortality losses associated with STEC disease in calves.

## 2. Results

### 2.1. Clinical Symptoms and Pathology

Three dairy production sites in Alberta experienced STEC disease outbreaks in their calf barns during the winter months of 2009–2011 (Table 1). These outbreaks occurred annually between December and February. According to the producers, these infections resulted in high annual morbidity and mortality losses (50% to 80%). In production site A, the calves either appeared normal at the last feeding time, but succumbed to disease within 2 h of the last visual inspection, or the calves developed progressive symptoms with an initial melana-like scour suggestive of upper intestinal hemorrhaging, labored breathing, followed by lying flat on their sides, wasting, grinding teeth and fur loss prior to death. In production site B and C, the calves also had progressive symptoms. Control calves (*n* = 3, data not shown) did not have any pathology or symptoms. Attempts at treating the calves with trivetrin, penicillin or tetracycline were ineffective. 

All tissues from calves colonized with O174 and/or O177 STEC co-infections presented with HE that included acute jejunal hemorrhaging, raised Peyer’s patches, severe focal hemorrhages, blood filled jejunal loops, mucosal erosions, dark-red erythema and edema. The hemorrhages, blood-filled distended loops and erythema were visible through the serosa. A milder focal hemorrhaging was present in the anterior cecum, but did not extend further into the colon. The ileum had no hemorrhaging but thick viscous yellow mucus was present which consisted of neutrophils, bacteria and cellular debris. In contrast, infections with O145 STEC dominant were associated with only the thick viscous yellow mucus throughout the intestinal tract consisting of neutrophils, bacteria and cellular sheets. We defined an STEC as an ExPEC as it infected peripheral organs including the liver, lungs and kidney. Infections with ExPEC dominant had the same pathology as O174 and/or O177 STEC infections. All infections entered the bloodstream as a severe complication and the changes in respiration were suggestive of septic shock.

**Table 1:** Clinical symptoms recorded for STEC-associated hemorrhagic enteritis (HE) cases in dairy calves from three production sites (A,B,C), jejunal hemorrhage syndrome (JHS) cases in older calves and mature cattle (D) and beef feeder calves from experimental O157 STEC challenge studies (E).

Site–Disease, serotype	Scour	Respiration	Appearance	Death
A–HE, O145 > ExPEC ^a^	after death: watery to white scour with no fecal matter and the presence of mucus and blood	normal	normal	mortality without warning
A–HE, O145 < ExPEC	dark brown feces with the presence of mucus and blood	normal changing to labored	depressed with drooping head, wasting, progressing to flat on its side	progressive
B–HE, O177	dark brown feces with the presence of mucus and blood	normal changing to labored	depressed with drooping head, wasting, progressing to flat on its side	progressive
C–HE, O174/O177	dark brown feces with the presence of mucus and blood	normal changing to labored	depressed with drooping head, wasting, progressing to flat on its side	progressive
D–JHS, (O157, O145, O177,O174, ExPEC)	grey-green feces	normal changing to labored	diarrhea, recumbent, wasting, hind end paralysis	progressive
E–HE, O157 (E318N, E32511N, H4420N, R508N)	runny feces	normal	normal	persistent shedding

^a^ Shiga toxin-producing *Escherichia coli* that cause disease outside the host intestinal tract.

### 2.2. STEC Co-infection

STEC disease outbreaks in humans have suggested that co-infection contributes to the development and severity of disease [18]. In the current study, STEC co-infections were present in the hemorrhaged and inflamed tissue (10^5^–10^6^ CFU/2.5 cm^2^) consisting of mauve with a white halo (O145) or blue with a mauve halo (ExPEC, O177, O174) colonies on CHROMagar™ O157. The isolates were confirmed as *E. coli* in the GN-ID A + B assay and produced Stx1 and Stx2 in the ImmunoCard STAT!^®^ EHEC test. Stx 1 and Stx 2 were also detected in the bloody digesta in the ImmunoCard STAT!^®^ EHEC test. As observed in other studies, the Stx2 expression was lost after only a few subcultures [19]. All infections in the calves consisted of two to three STECs of the same or different serotypes with one strain preferentially colonizing inflamed regions and the other strain colonizing eroded or hemorrhaged mucosa. For example, the ExPEC in production site A (10^6^ CFU/5 cm^2^ tissue) was dominant in the inflamed regions while the O145 STEC (10^5^ CFU/5 cm^2^ tissue) was dominant in the focal hemorrhages and Peyer’s patches. Interestingly, the ExPEC was almost absent from the tissue or digesta of the calves that succumbed to disease without warning in production site A. We previously reported STEC co-infections involved with HE in goats [20] and JHS in older calves and mature cattle [1,2]. The current study extends this list to include HE in calves less than one month old. It is not clear what advantage these mixtures have over individual STEC infections, but the consistency of occurrence suggests that the STECs derive a benefit from the interaction. 

### 2.3. Celmanax^®^/Dairyman’s Choice™ Application

**Figure 1:**
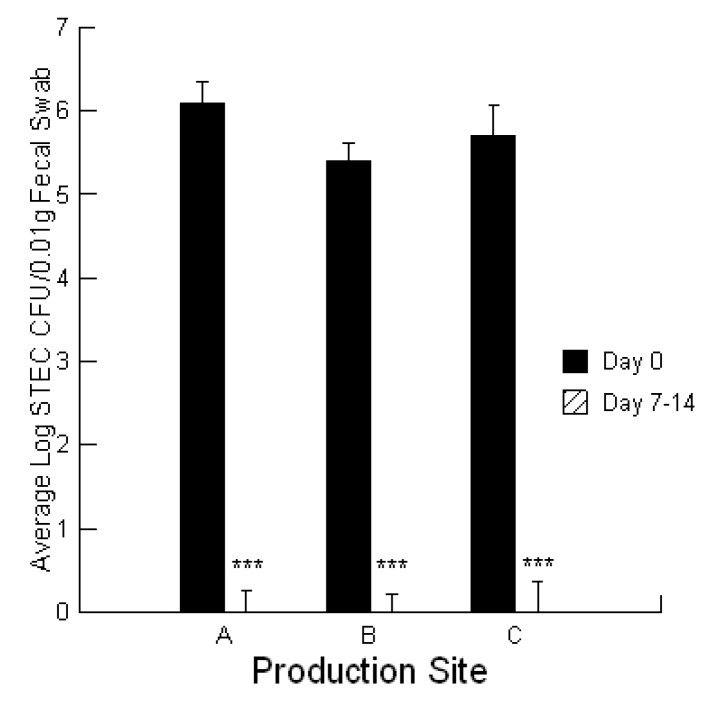
Effect of Celmanax^®^/Dairyman’s Choice™ applications on STEC shedding in calves from three production sites (A,B,C) at day 0 and day 7–14 (*n* = 3; *** *p =* 0.001).

Each production site reported 5 to 10 symptomatic calves all less than 4 weeks of age. The Celmanax^®^/Dairyman’s Choice™ application was 100% effective in eliminating symptomatic calves in 7 to 14 days (Figure 1, *p =* 0.001). The pattern for recovery of individual calves differed depending upon the severity of symptoms. Recovery from acute symptoms was progressive: first, upright sitting 24 h; second, standing after 48 h; third, walking at 72 h; fourth, bunting at 96 h; fifth, normal behavior at 120 h. Recovery from scours was progressive: first, stop scouring at 24 h; second, drier anal area at 48 h; third, no signs of scour 7 days. The producers reported no further health issues with the calves. The rapid decline in infection as evidenced by the STEC shedding pattern in the calves together with the loss of clinical symptoms suggest that this treatment was effective in assisting the defensive system in identifying and eliminating STEC infections even after the localized intestinal infection spread to peripheral organs.

### 2.4. Mycotoxigenic Fungi

Previous studies have indicated that the fungal spores identified in the feces of JHS cases predicted mycotoxin exposure [1,2]. There were no visible hyphae (400 times magnification; Nikon Diaphot inverted microscope) or fungal growth in the hemorrhaged tissues of the calves that developed HE. *Aspergillus flavus*, *Fusarium verticilliodes* and *Penicillium* *roqueforti* were present in the digesta of the hemorrhaged regions. There was no *A. fumigatus* present in the feed samples. There were several other types of mycotoxigenic fungi present in the commercial calf feed rations including *Aspergillus*, *Fusarium* and *Penicillium* species (Table 2). Interestingly, of the components found in the feed rations, the corn kernels were all positive for mycotoxigenic fungi. 

**Table 2:** Percent of calf feed ration collected from separate bags positive for mycotoxigenic fungi from three dairy production sites (A, B, C; *n* = 3).

Mycotoxigenic Fungi	A	B	C
*Fusarium verticillioides*	100	100	100
*Aspergillus flavus*	100	100	100
*Aspergillus versicolor*	100	0	0
*Penicillium roqueforti*	100	100	100
*Penicillium crustosum*	100	100	100
*Penicillium aurantiogrisium*	100	100	0

### 2.5. Mycotoxin Content of Feeds and Mucosa

**Figure 2:**
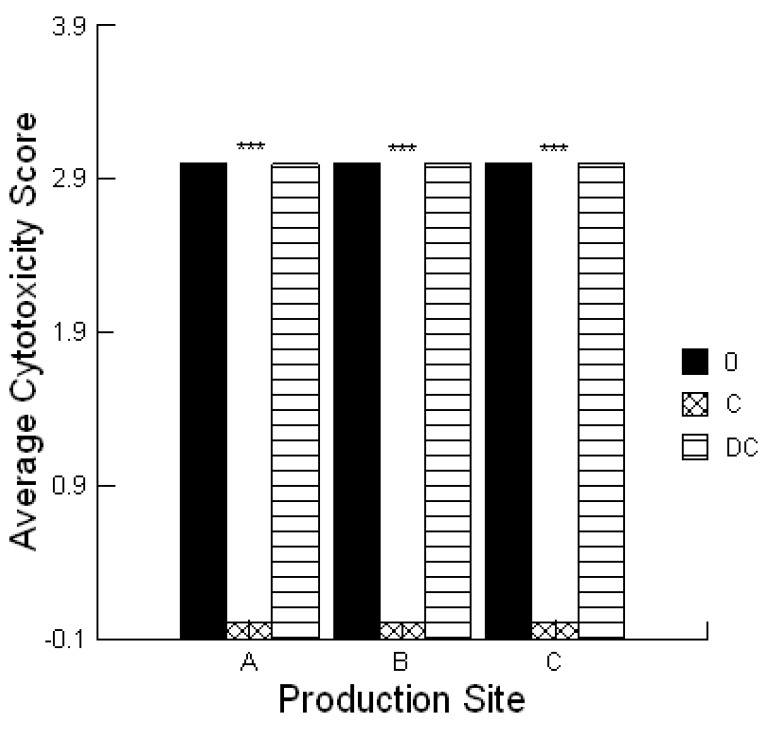
Impact of Celmanax^®^ and Dairyman’s Choice™ on the cytotoxicity of mycotoxin extracts from calf feed rations (*n* = 3; 0, extract alone; C, extract + 0.1% Celmanax^®^; DC, extract + 0.1% Dairyman’s Choice™ calf starter; *** *p =* 0.001).

For all production sites, the extracts from the calf feed rations had a high Cytotoxicity Score (3) or 100% cell death in the lawn assay. The Celmanax^®^ was 100% effective in preventing the cytotoxicity *in vitro* (Figure 2, *p =* 0.001) compared with the Dairyman’s Choice™ calf starter which had no effect. Analysis of the jejunal musoca from calves using the ELISA test strip method in association with a ROSA reader, confirmed the presence of aflatoxin and fumonisin (Table 3). DON, ZEAR, OCHRA or T-2/HT-2 toxins were not detected.

**Table 3:** Average concentration (ppb) of fumonisin and aflatoxin measured in the hemorrhaged jejunal mucosa of dairy calves using an ELISA-based method.

Dairy production site	Average Mycotoxin content (Mean ± SE)	
	Aflatoxin (ppb)	Fumonisin (ppb)
A (*n* = 2)	3 ± 0	50 ± 0
B (*n* = 2)	1 ± 0	350 ± 0
C (*n* = 2)	2 ± 0	250 ± 0
Control (*n* = 3)	0 ± 0	0 ± 0

The concentration of aflatoxin and fumonisin in the mucosa was the same amount as that detected in the digesta (data not shown) suggesting that there was a direct 100% transfer from the digesta to the mucosa. This is in agreement with reports for aflatoxin absorption by pigs and poultry, but is markedly different for fumonisin where 1%–6% absorption by pigs and poultry has been reported [21]. This may indicate that there was a higher level of fumonisin present in the feed, but it was not effectively extracted using the ROSA protocol. 

### 2.6. Calcium Response of Cells to Secreted Toxins from STECs Grown in the Absence and Presence of Fumonisin and Aflatoxin

**Figure 3:**
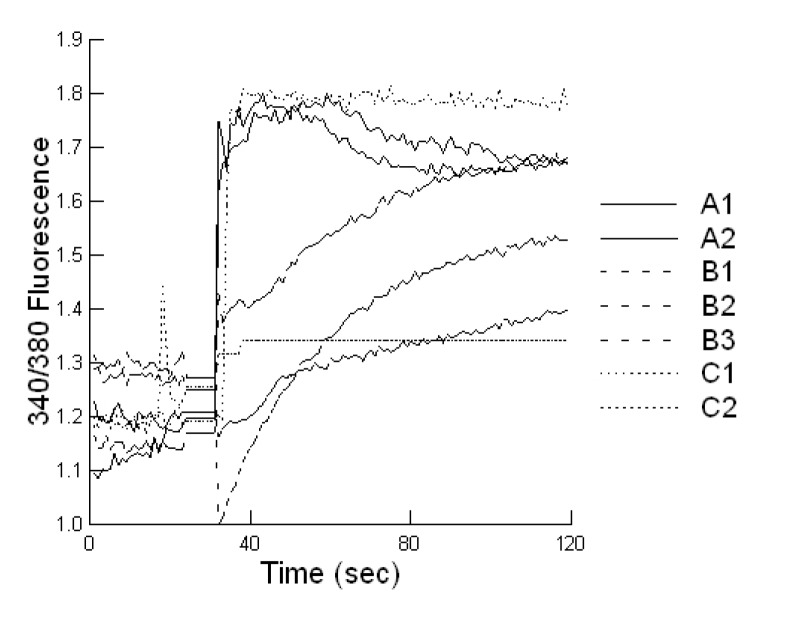
Effect of STEC-secreted protein composition on intracellular Ca^2+^ concentrations (340/380 fluorescence) in bovine liver cells. Ca^2+^ signaling in response to STEC-secreted proteins produced in M9 medium with normal 1 mM extracellular Ca^2+^ in the medium. The cells were loaded with fura-2/AM and stored in balanced salt solutions with or without Ca^2+^ to achieve a baseline before addition of the secreted proteins. Each value represents the Ca^2+^ mobilization for the protein composition secreted by STEC grown in M9 medium evoked in about 10 cells. Data are presented for each STEC involved in the infections at production site A (A1 = O145 STEC, A2 = ExPEC), B (B1–B3 = O177 STEC) and C (C1 = O177 STEC, C2 = O174 STEC). Recordings were performed at 37 °C and the experiment was repeated twice.

**Figure 4:**
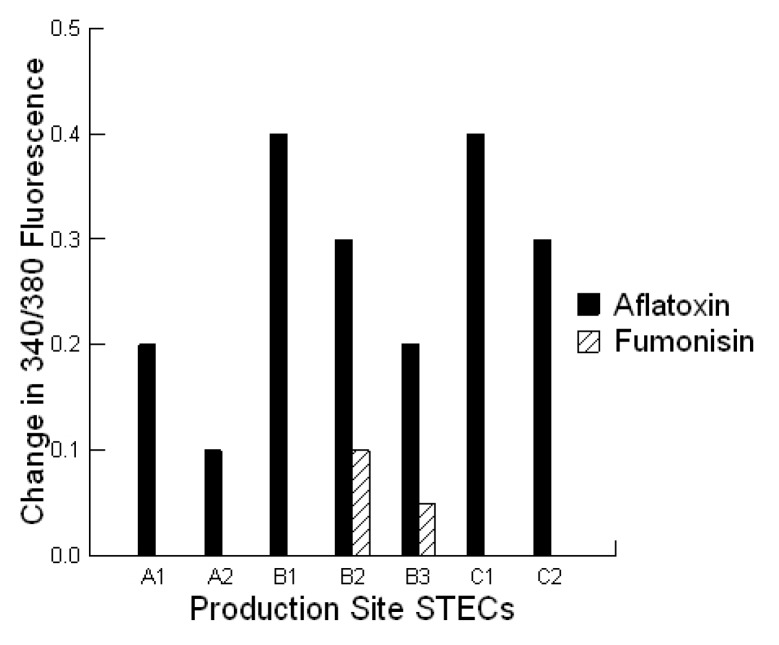
Effect of STEC-secreted protein composition on intracellular Ca^2+^ concentrations (340/380 fluorescence) in bovine liver cells. Ca^2+^ signaling in response to STEC-secreted proteins produced in the absence or presence of aflatoxin (0.02 ppb) or fumonisin (700 ppb) with normal 1 mM extracellular Ca^2+^ in the medium. The cells were loaded with fura-2/AM and stored in balanced salt solutions with or without Ca^2+^ to achieve a baseline before addition of the secreted proteins. Each value represents the difference in calcium mobilization of the mycotoxin-treated and untreated STEC-secreted protein composition evoked intracellular Ca^2+^ concentrations in about 10 cells. Data are presented for each STEC involved in the infections at production site A (A1 = O145 STEC, A2 = ExPEC), B (B1–B3 = O177 STEC) and C (C1 = O177 STEC, C2 = O174 STEC). Recordings were performed at 37 °C and the experiment was repeated twice.

Bacterial toxins can stimulate fluid secretion in the intestinal lumen through alterations in free cytosolic calcium [22]. This can result in sustained Ca^2+^ mobilization reflected as increases in intracellular Ca^2+^ concentrations (340/380 fluorescence) or in the case of pore-forming toxins, slow steady declines in intracellular Ca^2+^ concentrations. In this study, we exposed bovine liver cells to the secreted proteins from each STEC grown in M9 medium either alone or in combination with 0.02 ppb aflatoxin B1 or 700 ppb fumonisin B1. In both cases, the viability of the STECs was not altered by the presence of the mycotoxins (overnight growth = 10^9^ CFU/mL, data not shown). The intracellular Ca^2+^ concentration is usually kept at a very low level, around 100 nM in resting cells. Applications of toxins can increase cytosolic free Ca^2+^ 5 to 10-fold. There was no effect of 0.02 ppb aflatoxin or 700 ppb fumonisin alone on the intracellular Ca^2+^ concentration in the bovine liver cells. In the presence of external 1 mM Ca^2+^, the intracellular Ca^2+^ concentration of bovine liver cells was increased after the addition of the STEC-secreted proteins reaching a peak in 50 to 100 s (Figure 3; *p =* 0.001). At least one STEC in each group produced proteins that caused a sustained increase in intracellular Ca^2+^ concentration but below the maximum ratio achievable (Figure 3; *p =* 0.001). There were two types of calcium mobilization patterns: first, an instant and sustained increase in intracellular Ca^2+^ concentration; and second, a delayed and gradual increase in intracellular Ca^2+^ concentration . Regardless, a slow steady decline in the intracellular Ca^2+^ concentration was observed after 50 to 240 s for at least 1 STEC from each production site and continued to decline over a 60 min period. Addition of aflatoxin to the STEC growth medium produced a protein composition that provided a greater increase in the intracellular Ca^2+^ concentration compared with untreated growth medium (Figure 4; *p =* 0.001). In contrast, addition of fumonisin to the STEC growth medium produced a protein composition that provided fewer changes in the intracellular Ca^2+^concentration compared with untreated growth medium. Mycotoxin additions to the STEC growth medium produced a faster decline in the intracellular Ca^2+^ concentration for all STECs suggestive of an increased concentration of a pore-forming toxin (<50 s).

To validate this as a cytotoxin effect, we examined whether exposure to aflatoxin increased the amount of proteins produced by the STECs under the different growth conditions and applied the proteins (ng) to a lawn of bovine colonic cells to evaluate any changes in cytotoxicity *in vitro* (Table 4). This was measured as a threshold dose (ng) or the first concentration of protein produced by the STECs which elicited cell death. Although there was no consistent increase in the amount of protein secreted by the STECs (Table 4), all STECs grown with aflatoxin had a lower threshold dose for cytotoxicity (Table 4, *p =* 0.001) compared with STECs grown without aflatoxin. This suggests that aflatoxin changes the cytotoxin composition thereby increasing the Ca^2+^ mobilization or 340/380 fluorescence. Interestingly, at least one STEC in each co-infection (ExPEC, O177 STEC) produced a gelatinous growth medium in the presence of aflatoxin. Further studies are required to determine the nature of this material and its significance to colonization. Regardless of serotype, there was significant variation in aflatoxin-induced changes in cytotoxin composition as reflected in the increases in 340/380 fluorescence among STECs (*p =* 0.01). 

**Table 4:** The average production of secreted cytotoxins or proteins (ng/μL) by STECs and their associated threshold dose (ng) for cytotoxicity in a lawn assay using bovine colonic cells. Data are presented for STECs grown in the absence or presence of 0.02 ppb aflatoxin (*n* = 3).

STEC	Secreted proteins (ng/µL)	Average Threshold Dose (ng) ^a^
O145	15.34	11.1 ± 0a
O145 + aflatoxin	16.59	7.3 ± 1.9b
ExPEC	17.97	20.0 ± 4a
ExPEC+ aflatoxin	49.83	2.5 ± 0.5b
O177a	5.12	15.3 ± 0a
O177a + aflatoxin	5.44	8.1 ± 0b
O177b	17.05	25.5 ± 0a
O177b + aflatoxin	35.20	8.4 ± 2.1b
O177c	4.26	12.7 ± 0a
O177c + aflatoxin	5.68	7.1 ± 1.4b
O177	157.59	8.5 ± 3.2a
O177 + aflatoxin	130.08	6.0 ± 1.2b
O174	16.59	20.0 ± 4.0a
O174 + aflatoxin	11.72	7.1 ± 1.4b

^a^ column numbers followed by different letters are significantly different, *p =* 0.001.

In view of these results, we propose that aflatoxin exposure affects the STEC-secreted cytotoxin composition as reflected in the increasing intracellular Ca^2+^ concentrations with a corresponding increase in cytotoxicity. Further studies are required to determine the mechanisms underlying these changes.

### 2.7. Phenotype of Virulent and Avirulent STEC Strains

Modulation of the death pathways by STEC can inhibit or promote cell death through apoptosis, necrosis, and pyroptosis [23]. Pore-forming toxins such as Rtx initially increase intracellular Ca^2+^ concentration and then cause a slow steady decline in intracellular Ca^2+^ concentration after pore formation leading to cell death [24]. They may also cause biochemical changes in the cells that also lead to cell death [24]. Membrane blebbing is characteristic of apoptosis, while cell lysis or monolayer loss is characteristic of necrosis and pyroptosis. Exposure of bovine colonic cells to the secreted proteins from the virulent and avirulent STECs caused membrane blebbing with at least one strain in the STEC co-infections causing loss of monolayer integrity (Table 5). The avirulent E318N STEC caused cell blebbing without loss of monolayer integrity. The capability of STEC-secreted proteins from the virulent STECs to manipulate the necrosis and pyroptosis pathways suggests that STEC survival at infection sites and subsequent spread into the bloodstream may be associated with avoiding phagocytosis and clearance [25]. 

**Table 5:** Effect of virulent STEC and avirulent STEC (E318N) toxin mixtures on cell blebbing and monolayer viability. Modulation of cell death pathways for virulent STEC and avirulent O157 STEC (E318N) using a bovine colonic cell line (*n* = 3).

STEC	Cell Blebbing	Loss of Monolayer Integrity
E318N	3	
O145		3
ExPEC		3
O177a		3
O177b	3	
O177c		3
O177		3
O174		3

Diffuse and aggregative adherence have been linked to the presence of specific *nle* genes such as *nleH* with this feature promoting a rapid and more extensive colonization of cells [26]. The adherence patterns for the virulent STECs were diffuse or aggregative but localized for the avirulent E318N STEC supporting a role for these aggregative colonization patterns as promoting more serious STEC infections in cattle (Table 6). 

**Table 6:** Adherence pattern for virulent STEC from production sites (A, B, C) and avirulent O157 STEC (E318N) using a bovine colonic cell line (*n* = 3).

STEC	Diffuse	Aggregative	Localized
E318N			3
O145		3	
ExPEC		3	
O177a		3	
O177b	3		
O177c		3	
O177		3	
O174		3	

### 2.8. Virulence Gene Profile for STECs

In order to better understand the STEC infection process in cattle, we examined the differences between the virulence genes associated with natural disease outbreaks and experimental challenge studies. Using the genes determined to be relevant to high risk of severe human disease [27], we compared the virulent STECs associated with HE and JHS cases in calves with the avirulent O157 STECs (E318N, E32511N, H4420N, R508N) that were not causing symptoms in experimental challenge studies with beef feeder calves [5,28]. The results are presented in Table 7. The OI-122 encoded genes *nleB* (O103) and *nleB* (O157) were present in all virulent STECs but not in avirulent STECs. The OI-71 encoded gene *nleH* was associated with rapid development of disease and death in production site A. The OI-71 encoded *nleA* (EHEC) was present in both avirulent and virulent STECs; however, *nleA* (EPEC) was associated with STECs causing HE in calves less than one month old, but not for JHS cases in older calves or mature cattle. In contrast, the OI-71 encoded *nleF* and *nleG* genes were absent from avirulent STECs but were sometimes present in virulent STECs. The occurrence of the *nleB* genes in all virulent STECs suggests that these genes provide an advantage in competing with endogenous flora that could contribute to infections. The presence of the EHEC-plasmid pO157 and Stx-phage-associated genes in all STECs suggests that these genes, although relevant for development of infections, may not contribute to the severity of infection in cattle. Examining other virulence genes suggested that *bfpA*, *etpD*, *katP* were not associated with the development of severe symptoms or mortality losses in calves. In contrast, *espP* was associated with calf losses where there was no warning prior to death. 

**Table 7:** Comparison of virulence genes from STECs associated with mild to serious disease in immature and mature cattle.

Genetic Element ^a^	Clinical Symptoms					
	Virulence Gene ^b^	A (*n* = 2)	B (*n* = 4)	C (*n* = 2)	JHS cases (*n* = 5)	O157 Challenges (*n* = 4)
pMAR2	*bfpA*	-	-	-	-	3
pO157	*ehxA*	-	4	2	3	4
pO157	*espP*	2	-	-	-	4
pO157	*etpD*	-	-	2	2	4
pO157	*katP*	2	-	-	-	4
OI-71	*nleA(EHEC)*	-	4	2	2	4
OI-71	*nleA(EPEC)*	2	4	2	-	-
OI-71	*nleF*	-	-	2	2	-
OI-71	*nleH*	2	nd ^c^	nd	nd	nd
OI-122	*nleB(O103)*	2	4	-	2	-
OI-122	*nleB(O157)*	2	4	2	-	-
OI-122	*nleE*	-	4	2	2	-
OI-57	*nleG (O103)*	-	-	-	-	-
OI-57	*nleG (O157)*	2	-	2	-	-
OI-36	*nleD*	2	nd	nd	nd	nd
Stx-phage	*stx1*	2	4	2	3	2
Stx-phage	*stx2*	2	-	-	-	4
LEE	*eae*	2	4	2	5	4

^a^ pMAR2, Enteropathogenic *E. coli* (EPEC) adherence factor plasmid; pO157, O157 Enterohemorrhagic *E. coli* (EHEC) virulence plasmid; OI, O islands are unique DNA segments present in virulent *E. coli*. There are 177 O islands; OI-71, O island number 71; OI-122, O island number 122; OI-57, O island number 57; OI-36, O island number 36; Stx-phage, prophage encoded *stx* genes; LEE, Locus of Enterocyte Effacement Genes; ^b ^*bfpA*, bundle forming pili A; *ehxA*, hemolysin; *espP*, extracellular serine protease; *etpD*, type II secretion system; *katP*, catalase/peroxidase; *nleA* (EHEC) or *nle* (EPEC), an EHEC/EPEC translocated effector endoplasmic reticulum protein export inhibitor; *nleF*, apoptosis inhibitor; *nleH*, apoptosis inhibitor; *nleB*(O103) or *nleB* (O157) suppresses NF-κB activation; *nleE*, inhibits p65 nuclear translocation; *nleG* (O103) or *nleG* (O157), E3 ubiquitin ligases; *nleD*, prevents JNK-mediated pro-apoptotic signaling by cleaving and inactivating JNK; *stx1*, Shiga toxin 1; *stx2*, Shiga toxin 2; *eae*, intimin; ^c^ nd, no data.

One feature of the STEC disease outbreaks on all production sites was the ineffectiveness of antibiotics to alleviate disease. The STECs associated with HE cases had from 0 to 17 antibiotic resistance genes supporting antibiotic resistance as having a role in mediating infection and disease (Table 8). Antibiotics are administered in animal models to artificially reduce intestinal flora which enables studies of STEC pathogenicity [29,30]. In calf-rearing facilities, calves are given a prophylactic treatment of antibiotics at entry which could favor the development of infection and enhance the severity of infection.

**Table 8:** Antibiotic resistance associated gene profiles for STECs causing mild to serious symptoms in calves less than one month old.

Oligonucleotide Primer ^a^	A O145	A ExPEC	B O177a	B O177b	B O177c	B O177d	C O174	C O177
*70-aadA(1)290*	0	1	1	1	1	1	0	0
*70-aacC(2)200*	0	1	0	0	0	0	0	0
*70-aphA(1)1310*	0	1	0	0	0	0	0	0
*70-cat(3)370*	0	0	1	0	0	0	0	0
*70-dhfr(5)1560*	0	0	1	0	1	0	0	0
*70-dhfr(7)753*	0	0	1	1	0	1	0	0
*70-dhfr(9)830*	0	0	1	0	1	0	0	0
*70-oxa(7)295*	0	0	1	1	1	1	0	0
*70-sul(2)420*	0	1	0	0	0	0	0	0
*70-tem8674*	0	1	0	0	0	0	0	0
*70-tetB190*	0	1	1	1	1	1	0	0
*F_aph3strA*	0	1	1	0	0	0	0	0
*F_aph6*	0	0	1	0	0	0	0	0
*F_bla_SME1*	0	0	1	0	0	0	0	0
*F_bla_VIM2*	0	0	1	0	0	0	0	0
*F_cat*	0	0	1	1	1	1	0	0
*F_ereB*	0	0	1	1	1	1	0	0
*F_tnpM*	0	0	1	0	0	0	0	0
*RB_ereA2*	0	0	1	0	0	0	0	0
*F_mphA*	1	0	0	0	0	0	0	0
*RB_dhfrXII*	1	0	0	0	0	0	0	0

^a^ Resistance genes. *70-aadA(1)290*, streptomycin; *70-aacC(2)200*, gentamicin; *70-aphA(1)1310*, Kanamycin; *70-cat(3)370*, Chloramphenicol; *70-dhfr(5)1560*, Trimethoprim; *70-dhfr(7)753*, Trimethoprim; *70-dhfr(9)830*, Trimethoprim; *70-oxa(7)295*, β-lactamase; *70-sul(2)420*, Sulfonamide; *70-tem8674*, β-lactamase; *70-tetB190*, Tetracycline; *F_aph3strA*, Kanamycin; *F_aph6*, Kanamycin; *F_bla_SME1*, carbapenemases; *F_bla_VIM2*, carbapenemases; *F_cat*, Chloramphenicol; *F_ereB*, susceptibility to mercury compounds; *F_tnpM*, integrons associated with novel combinations of resistance genes; *RB_ereA2*, probe for macrolide resistance; *F_mphA*, probe for macrolide resistance; *RB_dhfrXII*, Trimethoprim.

Finally, expression of specific toxins such as RTX modulates cell death pathways allowing for manipulation of the host cell to promote STEC replication and dispersion within the intestinal tract [24]. All STEC co-infections in calves had the *ehxA* gene which is part of the RTX toxin gene family and either heat-stable or heat-labile enterotoxin genes (Table 9). In the membrane blebbing assay, we observed toxin activity as a ruffling of membranes while in monolayer loss, the central region of the cells expanded and stretched in length until cells ruptured. Since toxins enhance the survival of pathogens at infection sites, increase the potential for spread into the blood stream and promote colonization, the lack of clinical symptoms at one production site (A) prior to death may reflect the expression of a unique combination of toxins that readily promote host invasion. 

**Table 9:** Toxin gene profiles for STECs causing HE cases in calves less than one month old.

Oligonucleotide Primer ^a^	A O145	A ExPEC	B O177a	B O177b	B O177c	C O174	C O177
*70-astA(2)183*	0	1	0	1	1	1	1
*70-astA130*	0	1	1	1	1	1	1
*70-esta1365*	0	0	0	1	1	0	0
*70-hlyE867*	0	0	1	0	1	0	0
*70-stx1A742*	0	0	1	1	1	1	1
*70-stx1B1454*	0	0	1	1	1	1	1
*70-stx2A1087*	0	1	0	0	0	0	0
*70-stx2B(1)1353503*	0	1	0	0	0	0	0
*70-rtx586651*	1	0	0	0	0	0	0

^a^ Toxin genes. *70-astA(2)183*, Enteroaggregative *E. coli* heat-stable enterotoxin 1; *70-astA130*, Enteroaggregative *E. coli* heat-stable enterotoxin 1; *70-esta1365*, heat-stable enterotoxin I; *70-hlyE867*, silent hemolysin (haemolytic phenotype when overexpressed); *70-stx1A742*, Shiga toxin 1A; *70-stx1B1454*, Shiga toxin 1B, *70-stx2A1087*, Shiga toxin 2A; *70-stx2B(1)1353503*, Shiga toxin 2B; *70-rtx586651*, putative RTX family exoprotein.

## 3. Discussion

Many experimental challenge studies have been performed using O157 STEC and immature calves, particularly neonatal calves [3,4,28]. Generally, the infections are short-lived, as evidenced by a rapid decline in fecal shedding, and lack of clinical symptoms or pathology. There are exceptions where calves challenged with calf-scour origin O157 STEC have a faster onset of clinical symptoms and greater severity of disease compared with human-origin O157 STEC [3,4]. In these studies, calf mortality has been recorded. The virulence gene composition of the STECs used in early challenge studies were not well defined and limited to confirming key virulence genes such as *eae* and *stx*. In addition, the STEC origin was generally of human relevance and not chosen because it caused disease in cattle. To gain a better understanding of STEC pathogenicity in cattle, we initially compared the pathology found in the intestinal tract of highly infected or persistent shedding calves with low-infected or low shedding calves [5]. In these studies, persistent shedding calves that were shedding for five months developed a mild form of hemorrhagic enteritis while low shedding calves had no pathology. Interestingly, even the persistent shedding calves eventually resolved the STEC infections so that at five weeks after the last detectable shedding, only residual mucosal pathology remained in the intestine. Since no other experimental challenge studies had reported intestinal pathology, we interpreted these results as suggestive of unique STECs. One obvious difference in our studies was the use of four STECs of bovine and human origin instead of the single STEC of human origin used in almost all reported experimental challenge studies. We hypothesized that if STEC mixtures did promote infection, natural disease outbreaks should have STEC co-infections. We initially examined natural JHS or HE cases in immature and mature ruminants [1,2,20] and a minimum of two genetically distinct STECs were involved in the co-infections. In the current study, we further extend the significance of STEC co-infections to include HE cases in immature calves. Virulent STEC are able to colonize mucus more effectively in both the small and large intestine compared with less virulent STEC in the mouse model [29,30]. This higher level of colonization in the small intestine is associated with earlier presentation of clinical symptoms and increased severity of disease. In the current study, all HE cases for calves had high levels of STEC colonization of the small intestine equivalent to those reported for the mouse model supporting the small intestine as critical for persistent infection rather than the colon. Furthermore, the O145 STEC infections detected at one production site appeared to promote mucus formation compared with calves where the O145 STEC was not dominant. Therefore, the capability of rapidly colonizing via mucus may bring about more rapid systemic disease without any apparent pathology. As far as we are aware, no experimental STEC challenge studies with cattle have examined whether the infections can lead to bacteremia as they can in the mouse model. All HE cases with the calves developed bacteremia confirming earlier studies with JHS cases for cattle [1,2]. In contrast, beef feeder calves that were removed from experimental O157 STEC challenge studies due to persistent shedding presented with a milder form of HE, but did not have bacteremia [5]. Together, these results suggest that traditional experimental O157 STEC challenge studies may not have resulted in disease in older calves due to both the requirement for co-infections and the genetic nature of the STECs (*i.e.*, virulence gene composition) rather than an inability of this pathogen to cause disease. 

Antibiotic-treated, malnourished and germ-free mouse models have reduced normal intestinal flora that favors STEC colonization [31] and as such, are used extensively to characterize pathogenesis. These conditions are achieved in calves given prophylactic or therapeutic antibiotic treatments as preventative measures for disease [32], but also if the calves experience variable milk availability, milk quality or calf feed ration quality [33,34]. Once established, initial STEC infections may evolve into multiple clones having varied virulence composition as it is transmitted from calf to calf. This sequence for evolution of more virulent STECs from less virulent STECs has been described in a mouse model [29]. Inoculating mice with two types of STEC, EDL-933 (wild type) and EDL-933cu (cured of the 60-megadalton plasmid), resulted in two types of infections. The first type of infection, which represented 67% of the mice produced a typical co-infection with STEC EDL-933 high colonizing at about 10^7^ CFU/g feces for 16 days compared with STEC EDL-933cu that weakly colonized at about 10^3^ CFU/g feces for 3 days. The second type of infection, which represented 33% of the mice produced an atypical co-infection with STEC EDL-933 behaving as previously described, but with STEC EDL-933cu weakly colonizing at about 10^3^ CFU/g feces for 4 days and thereafter slowly increasing to 10^7^ CFU/g feces by day 11. Examination of the colonization sites suggested that the newly evolved STEC EDL-933cu recovered from the infected mice was better able to colonize the small intestine compared to STEC EDL-933 and the original STEC EDL-933cu. More significantly, inoculating mice with the newly evolved STEC EDL-933cu resulted in the development of earlier clinical symptoms and more serious disease. This new virulent STEC did not produce intestinal pathology, but did enter the blood stream damaging the kidneys. STEC clones are not a new observation for shedding beef calves in feed lots [35]; however, their relevance to persistence has been unclear. The current study suggests that the STEC clones from natural infections have evolved to produce more virulent STECs that are better able to colonize calves. 

Prebiotics and probiotics are registered for use in cattle diets to improve performance [36,37,38]. Initially studies were focused upon inclusion of these materials in diets to increase milk production, average daily gains and overall improved delivery of replacement stock. Reductions in unknown pathogen scours have been recorded for calves in other studies [38], but have for the most part given poor reductions in STEC shedding rates in beef feeder calves. Given the low infectious dose for STECs, it is imperative to identify cost-effective treatments to prevent infection and short-circuit transmission to foods. Over the past five years, the use of probiotics, prebiotics or synbiotics (prebiotic/probiotic combinations) to prevent and treat diarrheal diseases has gained support for human [39,40] and animal health [1,2]. The current study supports the use of a prebiotic/probiotic combination or synbiotic to eliminate STEC infections in calves. The mechanisms underlying prebiotic and probiotic actions are only now beginning to be elucidated. Probiotic actions include production of antimicrobial compounds, improving the host defense system, indirectly excluding pathogens and enhancing the barrier function of the intestinal lining [39,40]. In contrast, prebiotic actions include promoting the growth of beneficial bacteria and anti-adhesive behavior against pathogens. STEC infection is initiated after the STEC first adheres to the mucus and host cell surface, but then as the STEC replicates the bacteria are released and colonize sites downstream of the colonization site [29]. If adherence can be altered by the inclusion of a non-antibiotic treatment, then the subsequent infection process can be reduced or eliminated. Our earlier studies determined that the Celmanax^®^/Dairyman’s Choice™ application eliminated the clinical symptoms of O157 and non-O157 STEC challenges that resulted in JHS cases [1,2]. Celmanax^®^ liquid and Dairyman’s Choice™ dry had anti-adhesive properties against O157 and non-O157 STEC colonization of bovine cells *in vitro* thereby suggesting that this mechanism may in part be responsible for alleviating the clinical symptoms that lead to JHS cases. In addition, the ability of Celmanax^®^ to not only absorb mycotoxins as other materials such as clays do [41], but also to prevent interaction with cellular targets suggests that this mechanism may also in part be responsible for resolution of STEC infections. In this manner, the oligosaccharides present in prebiotics (Celmanax^®^) and a few probiotics (Dairyman’s Choice™) together with the distinct anti-adhesive properties of these materials may provide multiple pathways for mitigating STEC infections. 

Previous studies examining the impact of STEC-secreted toxins on the colonization of lineage 1 and lineage 2 O157 STECs suggested that the secreted toxins of lineage 1 STECs could increase lineage 2 colonization to the level of lineage 1 [12,42]. In the current study, we compared the cytotoxicity of the secreted toxins of STECs grown in the absence or presence of mycotoxins. Each STEC co-infection had 1 STEC that secreted toxins which produced a threshold dose (~10 ng) for cytotoxicity equivalent to a lineage 1 O157 STEC. The majority of the STECs associated with HE in calves produced a threshold dose (~20 ng) for cytotoxicity equivalent to a lineage 2 O157 STEC. After exposure to aflatoxin in the growth medium, the most significant change was the conversion of these threshold doses of secreted toxins to the level of a lineage 1 STEC. The STECs had enterotoxin and pore-forming toxin genes which both affect intracellular calcium and cell viability. The increase in intracellular calcium in response to the STEC-secreted toxins produced in the presence of the mycotoxins together with a proportional increase in cytotoxicity supports the increased expression of enterotoxins and pore-forming toxins. We did not identify specific toxins, but we did detect higher pore-forming activity after STECs were exposed to aflatoxins suggesting greater *ehxA* expression. Since intermediate lineage O157 STEC naturally express higher amounts of *ehxA* than the other lineages [42], it is possible that exposure of the STECs to mycotoxins could represent a pressure for evolution to intermediate lineages in STEC. Regardless, STEC co-infection would provide a toxin advantage to less virulent STECs allowing them to colonize more effectively. In addition, exposure to mycotoxins could directly or indirectly increase STEC colonization of the intestinal tract by increasing toxin expression.

Virulent EHEC and EPEC seropathotypes for humans can be distinguished from avirulent seropathotypes by the presence of the *nleB* gene or the aggregative/diffuse colonization pattern [26,27,43]. To gain insight into whether the severity of STEC infections in cattle could also be aligned with specific genes, we compared the prevalence of *bfpA*, *ehxA*, *espP*, *etpD*, *katP*, *nleA*(EHEC), *nleA*(EPEC), *nleF*, *nleH*, *nleB*(O103), *nleB*(O157), *nleE*, *nleG* (O103), *nleG* (O157), *nleD*, *stx1*, *stx2* and *eae* genes in natural disease outbreaks with experimental challenge studies. Virulent STEC infections in cattle are linked to the presence of *nleA* (EPEC), *nleF*, *nleH*, *nleB* (O103), *nleB* (O157), *nleE*, *nleG* (O157) and *nleD*. This alignment suggests that although the STECs associated with severe disease in cattle fall into different seropathotypes, they are closely related in terms of virulence genes. It is suspected that the ability to colonize in an aggregative or diffuse manner is associated with the *nle* genes which interfere with cell death and anti-inflammatory pathways [44]. For example, the recent O104:H4 STEC disease outbreak in Europe was not initially detected as it did not carry the genes for the traditional A/E lesion formation, but did carry the *nle* genes [45]. The aggregative and diffuse phenotypes observed for all virulent STECs in cattle together with their *nle* gene composition support these genes as indicators for serious forms of disease in cattle.

## 4. Experimental Section

The protocols were reviewed by the Agriculture and Agri-Food Canada Animal Care Committee and approved under ACC protocol 1131.

All animals developing acute symptoms were euthanized by the producer according to the codes of practice for the care and handling of dairy calves in Canada [46]. In 2009, two dairy calves underwent a *post mortem* examination to assess the pathology and to characterize pathogens except for production site A where two calves were examined for each type of clinical presentation. In 2009–2011, fecal STEC shedding was monitored before and after the Celmanax^®^/Dairyman’s Choice™ application. Feed components and jejunum tissue were also examined for mycotoxigenic fungi as described previously [1,2]. Control calves included calves that succumbed to urinary tract infections, difficult birth and bloat. 

### 4.1. Calf Health

All symptomatic calves (5 to 10 calves/production site) received a prebiotic (Celmanax^®^ liquid, 7 g/head/day) and a probiotic (Dairyman’s Choice™ calf starter, 7 g/head/day) application administered twice daily either orally between meals using a syringe for acute symptoms or within the milk or water for mild symptoms. The Celmanax^®^ consists of a non-living formulation of yeast cell walls or mannan oligosaccharide (MOS) and yeast metabolites. The Dairyman’s Choice™ calf starter (Animal Pro-Products, Arthur, ON, Canada) consists of protected live yeast, yeast metabolites, vitamins and minerals. The symptomatic calves also received a 10 cc application of Dairyman’s Choice™ paste at day 0. This paste consists of two *Bacillus* strains, yeast metabolites, vitamins and minerals. The Celmanax^®^ liquid and Dairyman’s Choice™ calf starter decrease O157 and non-O157 STEC colonization of bovine colonic cells *in vitro* and alleviate the development of JHS cases in mature cattle [1,2]. The Dairyman’s Choice™ paste does not affect STEC colonization *in vitro*. Finally, the prebiotic binds and prevents cytotoxicity by individual mycotoxins commonly tested as feed contaminants (AFLA, FUM, ZEAR, DON, T-2, OCHRA) *in vitro* [2].

### 4.2. Postmortems

Postmortems were performed on eight calves from three production sites using standard procedures. 

### 4.3. Pathogens

A 20 cm piece of tissue was removed from the acute hemorrhaged region of the jejunum and colon. The digesta and tissue were evaluated for STECs using a method previously described [1,2]. Released bacteria from tissue or digesta samples were stored at −80 °C in 25% glycerol-75% nutrient broth (Becton Dickinson, Oakville, Ontario, Canada) or were grown overnight at 37 °C in LB broth (Fisher Scientific, Ottawa, Ontario, Canada) when required. Samples were applied to CHROMagar™ O157 (Dalynn Biologicals, Calgary, Alberta, Canada). Presumptive STECs appeared as mauve colonies with a small to large white halo or blue colonies with a mauve halo. To confirm identity, the presumptive isolates were subjected to a GN-ID A + B biochemical test (Alere™ Canada, Ottawa, Ontario, Canada) and Stx expression was evaluated using an ImmunoCard STAT!^®^ EHEC test (Somagen, Edmonton, Alberta, Canada). To further characterize the composition of the infections associated with HE cases, distinct morphological colonies were examined in a DNA microarray (MaxiVir1.0) which carried 514 oligonucleotides of 70 bases in length targeting 348 virulence or virulence-related genes and 96 antimicrobial resistance or antimicrobial resistance-related genes found in gram-negative bacteria. 

To evaluate STEC burdens in the calves, fecal ESwab™ samples (Alere™, Ottawa, Ontario, Canada) were collected on day 0 of the treatment application for symptomatic calves and at day 7–14 for recovered calves. ESwab™ is a liquid-based multipurpose collection and transport system that maintains viability of aerobic, anaerobic and fastidious bacteria for up to 48 h.

Secreted proteins including cytotoxins can be encouraged in bacteria by using specific media that promote the process. For the STECs, each was grown in M9 media in the absence and presence of 0.02 ppb aflatoxin B1 and 700 ppb fumonisin B1 as described previously [42]. STEC-secreted cytotoxins were isolated and concentrated by using a combination of centrifugation and ultrafiltration. Briefly, the strains were grown in 10 mL of M9 media for 5 days. After growth, the cultures were centrifuged, and the supernatant containing the secreted cytotoxins was concentrated. The protein content of secreted cytotoxins was assessed by using a QuickStart Bradford protein assay kit (Bio-Rad Laboratories, Hercules, CA, USA). The concentrated complex of cytotoxins was then serially diluted in a 1:1 ratio with PBS to obtain final dosages that ranged from 1 to 30 ng/µL. These preparations can be used to confirm a dose-dependent toxicity and to determine the threshold dose (ng) where the first cytotoxicity occurs against bovine colonic cells [42]. 

### 4.4. Mycotoxigenic Fungi and Mycotoxins

The method used has been described previously [1,2]. For the isolation of mycotoxigenic fungi, a 10 g sub-sample of the calf feed ration was finely ground and a 5 mL volume added to a PDA plate. The plate was incubated for 1 to 7 days and the individual fungal isolates transferred to new PDA plates. *Fusarium* isolates were identified by examination of micro-morphological characters and by PCR amplification and sequencing of a fragment of the EF1-a gene and comparing the sequence with the FUSARIUM-ID database [47,48]. *Penicillium* isolates were identified by microscopic examination of morphology and by PCR using partial beta-tubulin sequences, Bt2a and Bt2b [49,50]. *Aspergillus* species were identified by microscopic examination of morphology [51]. 

For extraction of mycotoxins from the calf feed rations, a 25 mL aliquot of 50% methanol was added to 3 g of ground sub-sample of the calf feed ration and placed on a shaker at 200 rpm for 3 h. The supernatant was collected in another tube, and stored at 4 °C until use. The sample was then diluted and analyzed for AFL, FUM, ZEAR, DON, T-2 and OCHRA using ELISA test strips analyzed using the ROSA system (Charm Biosciences Inc., Lawrence, MA, USA) which we had previously found provided equivalent results to commercial HPLC methods except with fumonisin where it greatly underestimated the content [1,2]. 

For the mycotoxin content of calf feed rations, we followed the procedure described by the manufacturer (ROSA system, Charm Biosciences Inc., Lawrence, MA, USA) except that a known quantity of mycotoxin standard was added to each sample. This was necessary to ensure that the sample did not have materials present that affected detection. 

For measurement of the mucosal mycotoxin content, three 5 cm^2^ pieces of mucosal tissue was removed from the hemorrhaged region of the jejunum and extracted using the weight to volume ratios provided for extracting aflatoxin and fumonisin from grains. The negative control was mucosa from a control calf and the positive control was mucosa from the control calf with the addition of a known amount of aflatoxin or fumonisin standard.

The mycotoxin-absorbing properties of the prebiotic Celmanax^®^ liquid, and probiotic Dairyman’s Choice™ calf starter, were evaluated using a previously described lawn assay [1,2]. Briefly, 1% SeaKem^®^ agarose (Mandel Scientific, Guelph, Ontario, Canada) served as a support gel. Next, the lawn agarose (3 mL of 4% SeaPlaque^®^ agarose (Mandel Scientific, Guelph, ON, Canada)) was mixed with bovine colonic cells and poured over the support agarose. A 5 µL aliquot of the solvent used for the extraction process served as negative controls. Each extract (5 μL) was applied with or without 0.1% prebiotic or probiotic and the plate incubated for 4 h under standard culture conditions. The lawn was stained with 0.1% trypan blue (Sigma-Aldrich Canada, Oakville, Canada) and de-stained using PBS. Plates were scored the same day and the amount of cytotoxicity was scored as low (Cytotoxicity Score 1), moderate (Cytotoxicity Score 2) or high (Cytotoxicity Score 3) which was visualized as a faint blue spot, a blue spot or a dark blue spot, respectively. These activities were compared to two standards, ground corn containing 0.1 ppm aflatoxin that had a low Cytotoxicity Score (1) and 1 ppm aflatoxin that had a high Cytotoxicity Score (3). The assay was repeated a minimum of three times.

### 4.5. STEC Phenotype

The adherence pattern was determined for each STEC as previously described [52]. A bovine colonic cell line was prepared and grown at 37 °C in a humidified atmosphere of 5% CO_2_. To each well of a multi-well plate, 10^4^ cells were added in 2 mL of DMEM supplemented with 10% fetal bovine serum and 50 µg/mL gentamicin for 5 days or until cells were confluent. The medium in each well was then removed and replaced with DMEM. The STEC were grown overnight in nutrient broth at 37 °C. About 10^5^ bacteria were added to each cell monolayer in 2 mL of DMEM. The multi-wells were placed into a 5% CO_2_ incubator for 3 h at 37 °C. Then, each chamber was washed 3 times with pre-warmed PBS (pH 7.4) in order to remove non-adherent bacteria. Fresh DMEM was added to each chamber and incubated for another 3 h. Then, the multi-wells were washed 3 times with PBS, fixed with 70% methanol, and stained with 10% Giemsa prior to observation. Adherence was scored as localized, diffuse or aggregative. We defined the adherence patterns as: (1) localized, where micro-colonies attach to one or two small areas on the cells; (2) diffuse, where bacteria cover the cells uniformly; and (3) aggregative, where the bacteria have a characteristic stacked-brick-like arrangement on the surface of the cells and on the glass slide free from the cells.

The ability of the secreted cytotoxins to disrupt the cell monolayer integrity was evaluated using a method developed for *Listeria monocytogenes* [53]. Briefly, 1 µL of an overnight growth of each STEC was added to 10 mL of M9 media to promote optimal toxin expression and secretion [39]. After 5 days incubation at 37 °C, each bacterial suspension was centrifuged at 10,000 rpm and the supernatants passed through a 0.2 µm syringe filter. A 200 µL aliquot of the secreted toxins was added to each monolayer and placed into a 5% CO_2_ incubator for 4–24 h at 37 °C. If the monolayers were compromised at the assessment times, each chamber was washed 2 times with pre-warmed PBS (pH 7.4) in order to remove any detached cells. Then, the multi-wells were fixed with 70% methanol, and stained with 10% Giemsa prior to observation. Cell monolayer integrity was scored as positive for disruption of the monolayer where >50% of the monolayer was released and negative for no loss in monolayer integrity. Control treatments consisted of the M9 media alone. 

### 4.6. Calcium Response to STEC-Secreted Toxins

A bovine liver cell line was developed in our laboratory and maintained in DMEM supplemented with heat-inactivated fetal bovine serum (HyClone, Fisher Scientific Company, Ottawa, Canada) 10%, 50 µg/mL gentamicin. Cells were seeded in coverslip slides and after adhesion, cultures were washed three times with epithelial cell saline solution to remove non-adherent cells. 

Cells plated on coverslip slides (1000 cells/well), were loaded with 2 μM Fura-2-AM at 37 °C for 20 min, in epithelial cell saline (final volume 1000 µL). Slides were placed on an inverted microscope (Nikon Diaphot, Nikon Canada, Mississauga, Canada) equipped for cell population fluorescence measurements using a photometric detection system (Photon Technologies International, Ontario, Canada). The sample was alternatively illuminated (*t* = 10 samples per second) by monochromatic light (at 340 and 380 nm wavelengths), for 100 s after toxin exposure, through a × 40 oil immersion objective (Nikon Diaphot). Then additional 120 s exposures were taken every 10 min for 60 min. The emitted fluorescence was passed through a dichroic beam splitter, filtered and the signal captured by a photometric detector. For presentation, the fluorescent ratios (F340/F380) of treated cells were compared to untreated cells.

### 4.7. Lawn Assay for STEC-Secreted Proteins

A bovine colonic cell line was developed in our laboratory and maintained in DMEM supplemented with fetal bovine serum (HyClone, Thermo scientific, Canada) 10%, 50 µg/mL gentamicin. The lawn assay was used to compare the toxicity of secreted cytotoxins from the STECs to cells. The lawn assay was performed using the cytotoxins from STECs as described previously [39]. Briefly, a 1% SeaKem Agarose (Mandel Scientific, Guelph, Ontario, Canada) support gel was poured into a petri dish. Next, the lawn agarose (3 mL of 3.7% SeaPlaque agarose (Mandel Scientific, Guelph, Canada) was mixed with 3 mL of cell suspension and poured over the support agarose. Each toxin dilution (3 μL) was applied, and the treated lawn was incubated for 4 h under standard culture conditions. The amount of total cytotoxin applied was 3 µL of the range of threshold doses previously reported for lineage 1, lineage 2 and intermediate lineages STECs [39]. The lawn was stained with 0.1% trypan blue (Sigma-Aldrich) and de-stained using 1.84% KCl. Plates were scored the same day, and the amount of cytotoxin activity was defined as the threshold dose (ng) of secreted proteins in the dilution series to cause a blue spot on the lawn.

### 4.8. Statistical Analysis

Statistical analyses were conducted utilizing a repeated measures design for evaluating the impact of the prebiotic and probiotic application on STEC shedding (SYSTAT 10.2.01). Analysis was performed utilizing the Pearson Chi-square test to evaluate the differences in STEC toxins to cause cell blebbing and loss of monolayer integrity. This analysis was also performed to compare the activities of the mycotoxin extracts in the absence and presence of the prebiotic/probiotic treatment. An ANOVA was conducted to compare the calcium responses of the bovine liver cells to the secreted STEC toxins in the absence and presence of aflatoxin and fumonisin. Finally, an ANOVA was conducted to compare the cytotoxicity of the STEC-secreted toxins in the absence and presence of aflatoxin. 

## 5. Conclusions

The current study determined that STEC co-infections are associated with HE cases in calves less than one month old. Comparisons of virulent STECs with avirulent O157 STECs used in experimental challenge studies suggest that aggregative and diffuse patterns of adherence together with the ability to disrupt cell monolayers is critical for the development of serious disease. Exposing calves to 1–3 ppb aflatoxin and 50–350 ppb fumonisin in the calf feed ration promoted STEC-associated HE outbreaks. Inclusion of 0.02 ppb aflatoxin in the growth media of STECs resulted in greater cytotoxin production and cytotoxicity *in vitro* supporting a role for mycotoxins in STEC pathogenesis. The OI-122 encoded *nleB* gene was present in all virulent STEC, but not in avirulent STECs. The *nleA*, *nleD*, *nleE*, *nleF*, *nleG* genes were also associated with one or more of the virulent STEC co-infections. The *nleH* gene was uniquely associated with HE cases where there was no warning prior to calf death. The *eae*, *stx1* and *stx2* genes were present in all STECs suggesting that these genes may be required for initiation of infection, but not for the development of serious disease in cattle. Application of Celmanax^®^/Dairyman’s Choice™ to the calves eliminated STEC shedding and the morbidity/mortality losses. 
